# What Will the Others Do?

**Published:** 1896-06

**Authors:** 


### WHAT WILL THE OTHERS DO?

Another of the two-year schools places its name at the masthead of the school of ships now requiring a three-years' cruise of six months each on the waters of knowledge before its mark of proficiency will be granted to those who enlist under its colors. This step of the National Veterinary College will place it among the strong competitors for the future, and it will not lag in the race of recognition, for none have ever doubted the ability of its teachers or the excellence of its instruction; they only regretted that too short a time was afforded its matriculants to properly take advantage of the valuable instruction there afforded them. Its sincere friends and the tried and true advocates of higher veterinary education regretted, with a keen sense of sadness, that it was not a four-year school, or one where a post-graduate course was obtainable. It will now command a support that will add vastly to its growth and power, and it will realize at once that nothing will be lost from this advance step. It will wield a stronger power for good, to the general advancement of the profession than most others could do, be they ever so willing, and none will reap a greater direct reward than the school itself. Its location and associations, and the environments that have grown so strong and powerful in making Washington one of the great educational centres of the future, will add no little to its growth and development, and win for its projectors a high place among the roll of colleges. As predicted by this Journal many months ago, the cause of higher veterinary education will never again turn its face backward, and the future of the other two-year schools is now decided and determined. They may, for a time, face the current of popular opinion, and remain as they have been; but the gathering storm, fostered and suppressed amidst its own waters, will speedly wrest them from their unsafe moorings and engulf them in the waters of oblivion, forgotten and unmourned. Hasten the final battle.

The Detroit Veterinary College desires it to be known among the profession that they early contemplate the establishment of a three-years' course. Expensive buildings are in course of erection, to be ready for occupancy at the coming session in October next. A complete reorganization of the faculty will be consummated, and a more thorough course of instruction inaugurated. The Secretary of the Faculty says that the recent retirement of Professors Brenton and McEachran was not because of the short curriculum, but for other valid reasons.

